# Weakly Textured Objects Pose Estimation: A Comprehensive Review

**DOI:** 10.3390/s26123957

**Published:** 2026-06-22

**Authors:** Jialun Li, Fanwu Meng, Shiyang Mao, Wenhao Shu

**Affiliations:** School of Mechanical Engineering, Beijing Institute of Technology, Beijing 100081, China; 15081252316@163.com (J.L.); 3220240373@bit.edu.cn (S.M.); 3220240329@bit.edu.cn (W.S.)

**Keywords:** pose estimation, weak texture, deep learning, computer vision

## Abstract

Pose estimation is an important task in the field of machine vision, being widely used in robot grasping, augmented reality, and other applications. Weakly textured objects pose severe challenges due to scarce texture and low-density features, becoming a bottleneck in robot grasping. This paper systematically reviews recent progress in weakly textured object pose estimation, classifying methods into traditional and deep learning categories, and further dividing deep learning methods into instance-level, category-level, and unseen object-level. This review further summarizes the core issues of generalization limitations, real-time contradictions, and data bottlenecks in existing research. Combined with the practical needs of weakly textured scenes, the review points out that multimodal fusion optimization, lightweight model design, and low-cost annotation technology development are the future core research directions. The research results can provide a reference for algorithm design, experimental verification, and engineering applications in the field of weakly textured object pose estimation.

## 1. Introduction

Pose estimation refers to the technique of calculating the position and orientation of a target object in three-dimensional space relative to a reference coordinate system, using image or point cloud data. It serves as the core bridge connecting machine vision and the physical world [1,2,3]. The result of pose estimation is usually represented by six degrees of freedom (6DoF), comprising a translation vector (*T*) and pose parameters (Euler angles). In industrial assembly [4], robots rely on pose estimation to control the positioning accuracy of parts with an exceptionally low margin of error through pose estimation in order to complete precision assembly. In medical navigation [5], the pose estimation accuracy of surgical instruments directly affects the safety of minimally invasive surgery. In augmented reality (AR), accurate pose estimation of tiny objects enables the precise overlay of virtual objects onto the real world. In the field of cultural relic restoration [6], accurate alignment between virtual restoration models and physical artifacts can be achieved through pose estimation, providing millimeter-level guidance for restoration work. In autonomous driving [7], pose estimation of weakly textured traffic facilities, such as road guardrails and traffic cones, is a key prerequisite for ensuring the reliability of vehicle obstacle avoidance decisions and the safety.

Weakly textured objects are objects that lack obvious surface texture features making their pose difficult to estimate in images using traditional feature-point-based methods. The inherent characteristics of weakly textured objects bring multiple technical challenges to pose estimation. First, feature extraction is difficult. Traditional corner detection algorithms, such as Harris and Shi Tomasi [8], rely heavily on local pixel gradients. Consequently, they experience a critical drop in reliable keypoint extraction on weakly textured objects, resulting in difficulty in meeting the minimum sample requirements for pose calculation in terms of the number of feature matching pairs. The second issue is insufficient robustness. Phenomena such as directional shadows, low-light conditions, and specular highlights can obscure the object’s silhouette or alter apparent geometry, significantly degrading both rotation and translation accuracy. Third, there is a strong model dependency, as models based on geometric models require high-precision 3D model support. These challenges have led to pose estimation for weakly textured objects becoming a bottleneck problem in the field of machine vision and industrial automation [9]. Therefore, achieving technological breakthroughs has important practical significance for promoting automation upgrades in intelligent manufacturing, intelligent healthcare, and many other fields [10].

During the development stage of traditional methods, pose estimation focused primarily on manual feature design and geometric model constraints, evolving across three main technical branches. In the early days, manual feature-point matching methods served as the main approach. Lowe’s SIFT algorithm [11] achieved feature invariance through scale-space extremum detection, becoming a foundational tool for pose estimation of weakly textured objects. However, its feature repetition rate in texture-scarce areas was high. In the intermediate stage, the focus was on optimizing geometric models. The point-to-plane ICP algorithm proposed by Rusinkiewicz et al. [12] improved pose estimation accuracy by 20%, but it proved prone to converging on local optima when the initial pose deviation exceeds 10%. In the later stage, three-dimensional descriptor technology emerged, with Sameer et al. [13] proposing an incremental SFM method to achieve pose estimation in model-free scenes. However, its single-frame processing time exceeded 500 ms, which could not meet real-time requirements. The common bottleneck of all these traditional methods is the strong dependence on feature quality and prior knowledge, resulting in insufficient robustness in complex weakly textured scenes.

In the era of deep learning, with the breakthrough of convolutional neural networks in feature extraction, weakly textured object pose estimation has entered the data-driven era [14,15]. The object pose estimation methods based on deep learning can be classified into instance-level, category-level, and unseen-object methods according to their problem formulation [16]. In terms of instance-level pose estimation, which learns the specific features of single or multiple known instance objects through network learning, DeepIM, proposed by Li et al. [17], achieved sub-millimeter-level accuracy through iterative optimization, becoming a benchmark method for industrial scenarios. Category-level methods target objects of a certain category and achieve pose estimation for new individuals that have not been seen in that category by learning common features, thus solving the generalization deficiency of instance-level methods. MetaPose, proposed by Ben et al. [18], is based on a meta-learning framework that achieves few-shot category generalization and improves the pose estimation accuracy of new category objects by 45%. As a frontier direction, unseen-object methods target completely new categories or objects that have not been encountered during the training process. The recently proposed OP-Align [19] achieves pose estimation of weakly textured objects without annotations through self-supervised contrastive learning.

Despite significant progress in deep learning-based methods, there are still some core issues [20,21]. First, local gradients vanish [22]. On a weakly textured, homogeneous surface, the spatial gradient approaches zero, causing the network fail to generate the distinct feature vectors required for dense correspondence matching [23]. In addition, there is a receptive field ambiguity [24]. When a CNN’s receptive field slides over a smooth, uniform surface, the extracted local feature patches are practically identical. This creates a highly non-convex loss landscape, leading to severe ambiguity in translation and rotation predictions [25]. Furthermore, over-reliance on contour features constitutes a critical bottleneck. Because the internal surface yields no discriminative high-frequency activations, the network is forced to rely almost entirely on the object’s external boundary where the background provides contrast [26]. However, the contour of the weakly textured objects is implicit, making the network fail catastrophically. It is crucial to provide a comprehensive review of all relevant problem statements in order to enable readers to quickly understand the latest technological frontier of object pose estimation in this field.

Focusing on these core dimensions, this review covers the complete technical spectrum of pose estimation for weakly textured objects. It includes both traditional methods based on hand-craft features and geometric models and full-branch methods in the deep learning paradigm. Based on their generalization capabilities, these methodologies are systematically subdivided into instance-level, category-level, and unseen object-level categories. Furthermore, this review comprehensively examines various training paradigms, practical application domains, and benchmark datasets. This paper aims to clarify the most promising research directions to guide future efforts in the field.

**Figure 1:**
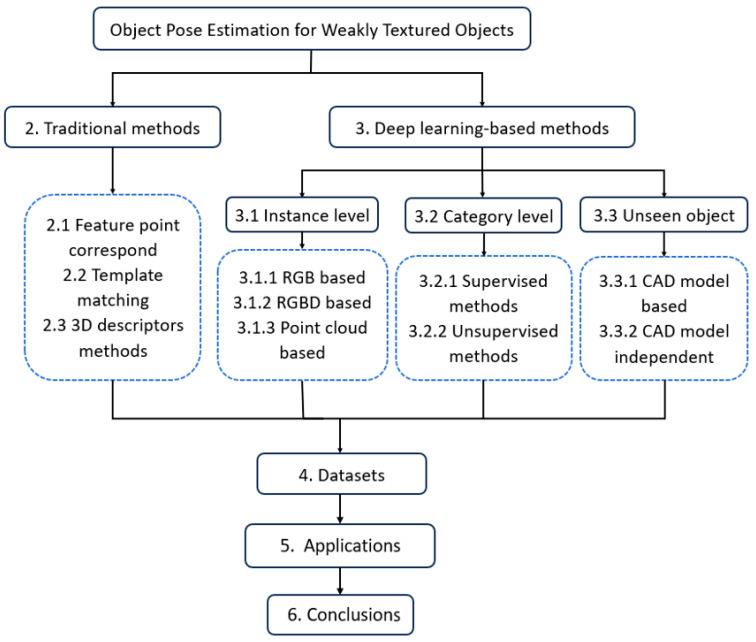
The structure of the review.

The structure of this article is arranged as follows. The first chapter clarifies the research value and analyzes the development of pose estimation. Section 2 introduces the traditional pose estimation methods. Section 3 introduces the learning-based pose estimation methods, analyzing the typical algorithms, and advantages and disadvantages of various methods. Section 4 is about datasets and metrics, quantifying the performance differences of various methods. Section 5 is about the applications and challenges, and Section 6 presents the conclusion and future directions. Figure 1 shows the structure of the article.

## 2. Traditional Pose Estimation Methods for Weakly Textured Objects

Before the era of deep learning, many methods based on manual features were designed for object pose estimating. Traditional pose estimation methods can be classified into three types: feature-point correspondence methods, template matching methods, and three-dimensional descriptor methods.

### 2.1. Method Based on Feature-Point Correspondence

This type of method is the fundamental technique for traditional visual pose estimation, which utilizes targeted feature-point extraction and matching strategies to mine the limited local features of weakly textured object surfaces. It is suitable for weakly textured scenes with a small number of distinguishable local features. The commonly used feature points include corner feature points, edge feature points, and speckle feature points.

The Edge Harris algorithm first identifies the effective area through Canny edge detection [27] and then extracts corner points near the edge, increasing the number of effective feature points of weakly textured objects by 50%. The ORB algorithm proposed by Rublee et al. [28] in 2011 combines FAST corner detection with binary optimization of the BRIEF descriptor to improve computational speed by two orders of magnitude while ensuring feature invariance. Its multi-scale edge fusion strategy increases the feature matching accuracy of weakly textured metal parts from 32% to 68%.

The edge feature-point extraction method based on Canny and Sobel edge detection is widely used to address the prominent edge information of weakly textured objects. The improved “Edge Chain Feature Point” algorithm [29] discretizes continuous edges into edge point chains and constructs descriptors based on the direction, length, and other information of the chains to solve the problem of single edge point matching ambiguity. The matching error rate on weakly textured objects such as ceramic vessels is reduced by 40%. Combining edge points with the “Edge-Normal” feature in the normal direction further enhances the discriminability of feature points in weakly textured scenes.

The speckle-based feature-point method is suitable for small grayscale variation areas on the surface of weakly textured objects, represented by the SURF and SIFT algorithms [30]. The SURF algorithm accelerates the calculation of Hessian matrix by integrating images to detect speckle features. Its improved version, SURF-64, improves the feature extraction efficiency on weakly textured plastic parts threefold by compressing the descriptor sub-dimensions.

### 2.2. Method Based on Template Matching

The template matching method constructs a multi-view template library for objects in advance, avoiding dependence on local feature points. It uses the global or local similarity measurement between the image and the template to achieve localization and pose solving, making it suitable for weakly textured scenes with extremely scarce feature points but complete edge and contour information. The main templates include grayscale templates and edge templates.

The grayscale template matching method uses normalized cross-correlation and sum of squared differences as the core similarity measure. The basic NCC algorithm [31] is susceptible to the influence of lighting uniformity in weakly textured scenes. The improved multi-scale NCC algorithm improves its adaptability to object scale changes by constructing a multi-scale template library, resulting in a large increase in positioning accuracy on weakly textured cylindrical components. The adaptive weight NCC algorithm assigns higher weights to edge regions, reducing the interference of grayscale uniform regions on matching results and improving the discriminability of similarity measures by 45%.

The edge template matching method focuses on the prominent characteristics of edge information and constructs an edge template library to achieve matching. The LINEMOD algorithm proposed by Hinterstoisser et al. [32] is a classic representative of this type of method. It constructs a template library by extracting multi-view edge features of objects and adopts a joint matching strategy of edge gradient direction and position. The improved Edge LINEMOD algorithm [32] introduces edge continuity constraints to solve the problem of template matching ambiguity in locally occluded scenes and can maintain a matching success rate of over 70%.

### 2.3. Method Based on 3D Descriptors

This type of method relies on the three-dimensional structural information of objects, such as point clouds, CAD models, or multi-view geometric constraints, and uses three-dimensional operators to achieve three-dimensional to two-dimensional or three-dimensional matching and pose solving. The core advantage is that it completely eliminates texture dependence and is suitable for weak texture scenes with three-dimensional models or obtainable multi-view sequences.

The method based on ICP and its derivative operators is a classic operator for 3D point cloud matching. It was proposed by Besl in 1992 [33] and iteratively searches for the nearest point between two sets of point clouds, optimizing the pose. However, in weakly textured scenes, it is prone to getting stuck in local optima due to sparse point clouds. The point-to-plane ICP proposed by Rusinkiewicz et al. [12] changes the matching constraint from point-to-point to point-to-plane and improves the pose estimation accuracy of weakly textured objects by 20%–30% using the model surface normal vector. In response to the sensitivity issue of the initial pose, the improved two-stage ICP of coarse matching and fine matching first obtains the coarse pose through contour matching, and then performs ICP fine optimization, increasing the convergence rate a lot.

The SfM (Structure from Motion) uses multi-view sequences to recover 3D structures and poses. Furukawa et al.’s [19] incremental SfM introduces motion constraints in weakly textured scenes, controlling the cumulative error of pose estimation within 5%. Global SfM [34] improves pose accuracy by 30% on weakly textured cultural relic image sequences by solving all camera poses at once, avoiding incremental error propagation. Combining IMU with VIO such as OKVIS [35], the interruption time for localization in weakly textured tunnel scenes has been reduced from 5 s to 0.5 s.

The traditional methods for pose estimation are essentially a geometric optimization problem, calculated based on manually designed features, with high interpretability. These traditional methods can play a certain role when facing simple and ordinary scenarios, but they have shortcomings in accuracy and robustness when facing complex scenarios. Gradually, these methods have now been replaced by data-driven deep learning methods that utilize deep neural networks to learn high-dimensional feature representations, thereby improving accuracy and robustness to complex environments.

## 3. Deep Learning-Based Pose Estimation Method

### 3.1. Instance-Level Methods

The instance-level pose estimation method is the fundamental application form of deep learning in the field of object pose estimation. Its core localization is to target a single or multiple predefined known instance objects, learn the feature representation specific to that instance through deep neural networks, and achieve accurate pose calculation. It cannot be directly transferred to other untrained objects. Based on the differences in core technology paths, instance-level pose estimation methods can be divided into three categories: RGB-based methods, RGB-D-based methods, and point cloud methods.

#### 3.1.1. RGB-Based Methods

Methods based on RGB image input rely only on two-dimensional color images and usually follow one of two technical routes, 2D-3D correspondence with PnP [36] or rendering-based comparison. The core challenge of these types of methods is to extract effective discriminative features from weak texture regions with smooth grayscale changes. Therefore, researchers often adopt multi-scale feature fusion and edge enhancement strategies. PoseCNN [37], as a pioneering algorithm in this field, uses a fully convolutional network to simultaneously predict object bounding boxes, semantic masks, and 2D coordinates of key points. After selecting key points in the target object area, combined with the 3D model coordinates of key points, the PnP algorithm is used to solve the pose. Its multi-scale feature fusion module enhances the edge response of weakly textured areas, reducing the pose estimation error of objects by 30%. This method performs outstandingly in complex scenes such as weak texture, symmetry, and occlusion.

DeepIM [17] innovatively introduces an iterative optimization mechanism based on PoseCNN, with the core goal of solving the problems of large initial pose prediction errors and high ambiguity in keypoint matching in weakly textured scenes. DeepIM effectively alleviates the problem of insufficient feature points for weakly textured objects by supplementing structural information with virtual images. Its iterative optimization mechanism gradually approaches the optimal solution, thereby improving pose estimation accuracy. Experimental data show that on the T-LESS dataset, DeepIM can control the translation error of typical weakly textured objects, such as bolts and bearings, to within 0.08 mm, which improves the accuracy by more than 40%. However, DeepIM does not directly perform pose detection, but rather significantly improves the accuracy of rough estimation results, meaning that it is heavily reliant on the initial pose.

The DPOD proposed by Shugurov et al. [38] decouples instance segmentation and dense coordinate regression into two steps: First, Mask R-CNN provides pixel-level object masks, and then a fully convolutional network is used to regress the corresponding 3D model coordinates and uncertainty pixel by pixel within the mask region. After obtaining the dense 2D-3D correspondence, RANSAC PnP is used to solve the 6D pose, and the uncertainty is weighted into the error function, significantly reducing interference caused by occlusion or blurred boundary points. This method corresponds to dense natural processing of rotational symmetry without pose ambiguity, but its robustness under extreme occlusion is not as good as that of PoseCNN.

INeRF, proposed by Liu et al. [39], takes the opposite approach. Rather than training the network to predict 2D-3D correspondences, it formulates pose estimation as the inverse problem of NeRF. Given an RGB query image and a pretrained NeRF scene or object model, a differentiable rendering is used to synthesize images from the current 6D pose assumption, and the SE pose is directly optimized using gradient descent by minimizing the photometric error between the synthesized image and the query image. To avoid rendering NeRF images excessively heavy, the author proposes region-of-interest sampling, which can converge with only about 1/100 pixel. Experiments have shown that INeRF could gradually refine the rough initial error in real scenes, and can use the estimated new views to enhance NeRF training in reverse. This method does not require CAD models and has extremely high accuracy.

Peng et al. [40] designed PVNet to address occlusion and truncation issues. This method innovatively introduces the projection vector mechanism to project three-dimensional keypoints onto a two-dimensional image plane, achieving precise cross-modal alignment of RGB features, effectively solving the problem of cross-modal feature misalignment in weakly textured scenes and further improving matching stability. In the RGB unimodal scene, its technical core can be summarized into three modules: 3D keypoint projection, 2D feature encoding, and spatial relationship constraint. Its advantage lies in the lack of iterative optimization, with a single frame processing time of about 50 ms, balancing accuracy and efficiency. The limitation is the strong dependence on the 3D model of the object, and the preset number and position of 3D keypoints will affect the estimation accuracy.

Su et al. [41] proposed ZebraPose, which treats the surface of an object as a zebra-stripe pattern. By dividing the surface into 2*^N^* stripes through *N* binary iterations, each stripe is uniquely identified by an *N*-bit binary code. To establish dense 2D-3D correspondences, the network only needs to predict pixel-level stripe encodings and foreground masks from a single RGB image, subsequently using Progressive-X PnP to solve the 6D pose in a single step. Thanks to its discrete and hierarchical encoding space, the network successfully maintains sub-pixel-level localization accuracy even for weakly textured, symmetric, or thin-walled objects. On the T-LESS, LM-O, and other datasets of the BOP benchmark, ZebraPose ranks among the top three using only the RGB modality, and its training data can be generated entirely from synthetic images without relying on real-world depth data. Despite successfully synthesizing data for training, the approach strictly relies on having an exact 3D CAD model of the object, and the data synthesis and subsequent training processes incur significant computational expenses.

#### 3.1.2. RGB-D-Based Methods

The RGB-D input method combines the appearance features of RGB images with the geometric features of depth images and enhances the feature expression of weakly textured objects through cross-modal information complementarity, balancing discriminability and robustness. It is currently the mainstream solution for instance-level weakly textured pose estimation.

Li et al. [42] proposed DenseFusion, a classic representative of this class of methods. It maps RGB and depth features into a shared feature space through a pixel-by-pixel dense fusion strategy, effectively enhancing the feature responses of weakly textured regions. Experiments results demonstrate that DenseFusion achieves an accuracy of 93.1% on the ADD(S) metric of the YCB-Video dataset. Furthermore, it maintains an 88% detection rate even in the heavily occluded dataset. The model operates in real time, making it highly suitable for precise robotic grasping tasks, but the overall performance of the model is heavily reliant on the accuracy and quality of the input depth data.

Co-Fusion [43] treats multi-frame RGB-D as a temporal point cloud stream, maintains an online object model using a global voxel grid, and simultaneously detects new frame instances with Mask R-CNN. Each frame performs 6D registration between the current observed point cloud and the model, and then renders the updated model into pseudo-depth and observed depth for joint photometric-geometric optimization, achieving joint tracking and reconstruction frame by frame. This method is the first to unify real-time object reconstruction and instance-level 6D tracking into a differentiable framework, supporting simultaneous updates of multiple objects in dynamic scenes.

**Figure 2:**
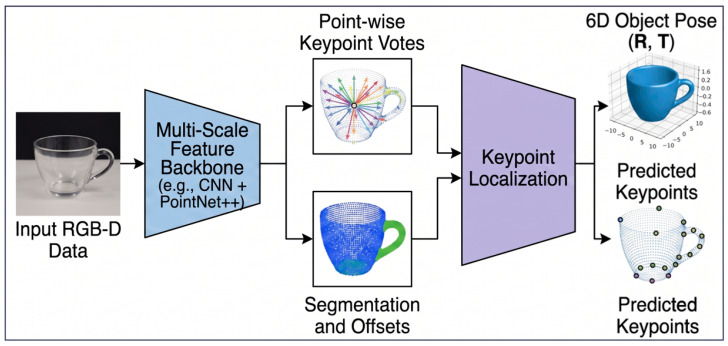
The structure of PVN3D.

He et al. [44] proposed PVN3D in 2021, which extends the 2D keypoint voting concept into 3D space. As illustrated in Figure 2, the network extracts features from the entire depth point cloud using PointNet++. Following a point-wise concatenation with RGB features extracted by a CNN, the model predicts a unit vector for each foreground pixel pointing toward one of eight predefined 3D keypoints. The 3D positions of these keypoints are obtained through depth Hough voting, followed by least squares fitting. The primary contributions of PVN3D lie in its high stability, fast training speeds, and ease of convergence. With an ADD(S) score of 92.5% on YCB-Video, it has become an important baseline for subsequent RGB-D methods. However, a notable limitation of this approach is its heavy reliance on an external instance segmentation module to accurately isolate foreground pixels. In the same year, they proposed the FFB6D full-flow bidirectional fusion network, which exchanges RGB and depth information back and forth in each layer of the network, rather than performing early or late fusion only at the end. This significantly improves the ability to distinguish occluded and low-texture areas. PVN3D is stable, easy to converge, and fast in training, but it relies on instance segmentation. This structural innovation serves as a major advantage, significantly enhancing the model’s ability to distinguish features in heavily occluded and weakly textured regions.

Transpose, proposed by Chen et al. [45], is the first to introduce a pure Transformer structure into RGB-D 6D pose estimation. The encoder flattens 2D pixel features and 3D point cloud features into a unified token, achieving cross-modal global association through self-attention. The decoder outputs N instance queries, with each query directly regressing the 3D center and 6D pose. This method is suitable for high-precision demand scenarios, but has poor real-time performance and requires multiple iterations.

The SAM-6D proposed by Lin et al. [46] utilizes the Segment-Anything Model for zero-shot segmentation of RGB images to obtain instance masks. After back-projecting to generate target point clouds, a two-stage coarse-to-fine Iterative Closest Point (ICP) is performed with the CAD model point clouds to complete 6D pose estimation. The entire process requires no retraining or fine-tuning, and proposes adaptive keypoint scoring based on normal-color consistency, effectively suppressing occlusion and background interference. On the 24 test sets of the BOP Challenge 2024, the average recall reaches 0.89, which is 2.4% higher than the second place (requiring training). While the method is inherently constrained by its reliance on exact CAD models and operates with a moderate inference time of 120 ms per frame, its robust framework provides a highly promising new paradigm for zero-shot pose estimation.

#### 3.1.3. Point Cloud Registration Methods

With the widespread adoption of 3D sensors, estimating the 6D pose of known objects directly using point clouds as input has become a research hotspot. Local features of the target are extracted from the point cloud, and then within the scene point cloud features, the extracted features are matched with pre-extracted template features through querying to obtain the 6D pose of the object. The pure point cloud method is not affected by lighting and texture loss, and is more robust to reflective objects.

Sarode et al. [47] proposed PCRNet, which simultaneously processes both the source point cloud and the template (CAD) point cloud through a shared PointNet encoder. The extracted 1024-dimensional global features are then concatenated and passed through three fully connected layers to directly regress the pose. Millisecond-level registration can be achieved without 3D-3D correspondence, relying solely on global feature regression. The single forward design enables the network to complete inference within 15 ms, providing possibilities for real-time robot grasping. On the LineMod point cloud dataset, ADD is slightly lower than the voting-based method, but the speed is increased by fourfold. Overall, PCRNet serves as a minimalist and lightweight baseline whose major advantage is its exceptional ease of deployment; however, this efficiency comes with a significant drawback, as the model exhibits weak robustness against occlusions.

In 2020, Chen et al. [48] proposed PointPoseNet, which takes the original point cloud of RGB-D back projection as input and outputs the 6D pose of the instance. The network is executed in parallel on the basis of PointNet. It is the first to fully transfer the idea of “2D keypoint voting” to 3D point cloud space, avoiding the loss of geometric details caused by voxelization. Unit vector regression makes the network insensitive to occlusion. This method belongs to the category of high accuracy and strong anti-occlusion in pure point cloud methods, but its speed is relatively slow.

Yuan et al. [49] viewed point cloud registration as an optimal transportation problem between two Gaussian mixture distributions. They were the first to combine “depth features-probability model-closed-form solution”, eliminating the need for iteration and correspondence search. This approach exhibits theoretical robustness against local missing data and uneven density. This method is applicable to scenarios that require only global registration and no initial values.

PS6D (Point-Cloud Segmentation 6D) proposed by Yang et al. [50] in 2024 integrates instance segmentation and pose estimation into a point cloud Transformer framework, avoiding the coupling of external 2D detection errors. It proposes density-adaptive voting to balance the weights of sparse and dense regions. However, a notable limitation of this approach is its strict reliance on the quality of the input point clouds, meaning that its performance can be compromised by poor raw 3D data. Finally, Table 1 summarizes the performance of this and other representative instance-level methods across the LM, LM-O, and YCB-V datasets.

### 3.2. Category-Level Methods

Category-level pose estimation aims to estimate the 6D pose of an unseen object instance without requiring its exact CAD model, provided only that the object belongs to a known category. The core breakthrough lies in its ability to overcome the “one-to-one” generalization limitations of instance-level methods. The core strategy of this type of method for weakly textured scenes is to explore generic geometric structural features within the category, avoiding dependence on individual texture features. Based on the reliance on training data, category-level 6D pose estimation methods can be strictly divided into two major categories: supervised methods and unsupervised/self-supervised methods.

#### 3.2.1. Supervised Methods (Require 6D Annotation or NOCS Labels)

The supervised category-level methods learn the mapping relationship from image or point cloud features to category-common poses by utilizing labeled samples within the category, where each training sample has a known label or output value. Its core advantage lies in its ability to accurately capture category-specific geometric structures and enhance the robustness of pose estimation by emphasizing common geometric features in low-texture scenes.

**Figure 3:**
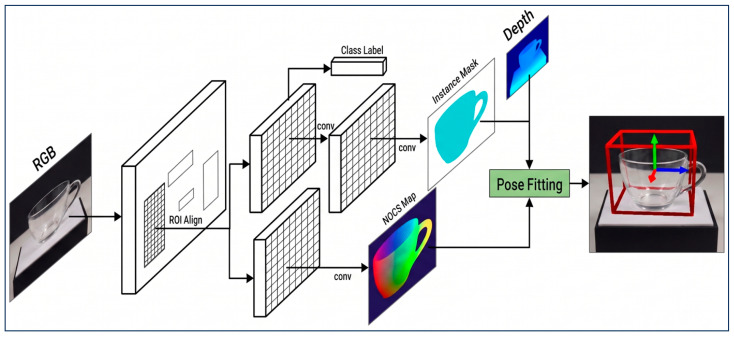
NOCS (Normalized Object Coordinate Space) map.

The Normalized Object Coordinate Space (NOCS) proposed by Wang et al. [51] utilizes CNN to predict normalized coordinate maps, which are aligned with the deep point cloud to obtain 6D dimensions. It is the first to introduce the category-level 6D concept and establish the NOCS benchmark. The network structure is built upon the Mask R-CNN framework, with the addition of a branch for predicting NOCS maps. The CNN predicts object category labels, masks, and NOCS maps from RGB images, and then fits the NOCS maps with the depth maps to obtain the 6D pose of the object. Figure 3 shows the structure of the NOCS network.

Zheng et al. [52] proposed HS-Pose, which adopts a 3D-GC hybrid encoding for category-geometry, and verified the effectiveness of the “category-geometry” hybrid convolution. The HS layer extends 3D-GC to extract hybrid scope latent features from point cloud data for category-level object pose estimation tasks. The ADD-S on Wild6D increased by another 4%. This method is applicable only at the category-level and relies on point cloud segmentation.

The mentioned methods are all based on shape priors, utilizing a pre-trained shape prior as an auxiliary. Liu et al. [53] proposed IST-Net, an efficient and concise pose estimator. It aims to achieve the transformation from the camera coordinate system to the world coordinate system at the feature-level, while abandoning the use of priors. When facing new categories, the reliance of prior-based methods on priors results in weaker generalization capabilities. However, IST-Net demonstrates significantly better generalization performance when dealing with relatively simple objects.

Zhang et al. [54] proposed GenPose, which innovatively redefines category-level pose estimation as a conditional distribution modeling problem. Within this framework, a generative network simultaneously outputs shape codes and pose distributions, while the energy-based model selects the optimal hypothesis. By pioneering this novel “generate-and-filter” paradigm, GenPose makes a profound contribution to the field, successfully achieving state-of-the-art performance. Furthermore, a major advantage of this probabilistic generation approach is its exceptional robustness and suitability for handling highly symmetric objects. However, a notable drawback of this sophisticated architecture is its relatively slow inference speed.

ASD-Pose, proposed by Tang et al. [55], takes adaptive reconstruction from sparse point cloud to dense point cloud as its core idea, addressing three major challenges: large geometric differences within similar objects, poor Mask R-CNN segmentation [56], and inaccurate sparse correspondence. It uses dense 3D-3D correspondence for one-time SVD rough estimation and ICP refinement to obtain the accurate pose. This method is suitable for visual localization of multiple objects mixed in industrial assembly lines, with high accuracy but average speed.

#### 3.2.2. Unsupervised/Self-Supervised Methods

Unsupervised category-level pose estimation refers to a paradigm that completely eliminates the need for ground-truth 6D poses or NOCS labels during the training stage. Instead, these methods rely solely on geometric consistency of RGB-D sequences, self-supervised reconstruction loss, or 2D/3D basic model priors. By leveraging these alternative cues, the network learns to map unseen objects of a given category from the observation space into a normalized coordinate system, thereby directly predicting their full 6D poses and metric dimensions.

Chen et al. [57] proposed ZeroPose, which pioneered the extreme application of “zero 6D labels”. It learns solely within the pre-trained CLIP space of a vast number of image–text pairs. Upon inputting a single RGB image, the network first predicts normalized coordinate maps, and then automatically selects the most matching “virtual template” using image–text similarity. It directly outputs category-level poses through differentiable PnP. This method lays the ideological foundation for subsequent zero-shot pose research.

**Figure 4:**
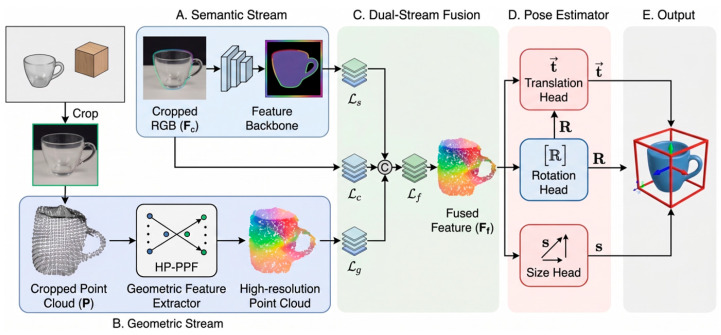
The architecture of SecondPose.

Chen et al. [58] pointed out that intra-class shape variations frequently lead to feature drift. To address this, they proposed SecondPose, an innovative “SE (3)-consistent dual-stream” architecture. As illustrated in Figure 4, one stream of the network is dedicated to extracting DINOv2 semantic features, while the parallel stream extracts self-supervised geometric features. During training, the two streams of features are explicitly constrained to the same rotation–translation manifold. At inference time, the network can regress the 6D pose of a new instance of the same class with only one forward pass. Furthermore, this dual-stream approach demonstrates robust generalization, making it highly effective in environments characterized by rich textures and diverse shape variations.

FoundPose [59] introduces an approach that completely freezes the underlying pre-trained foundation models. It extracts 2D features using DINOv2 and 3D features using self-supervised FCGFs. The two establish 2D-3D correspondence through cross-modal optimal transport, and then solve for the pose using PnP. The most profound contribution and advantage of this method is its truly “out-of-the-box” zero-shot capability, as the entire pipeline operates completely without the need for fine-tuning or 6D pose labels. On the LineMod dataset, it achieves an ADD of 97.2%, approaching the fully supervised level for the first time with zero-shot results. Thanks to its robust generalization capabilities, FoundPose proves to be highly advantageous for the rapid validation of novel objects and small-batch industrial deployments. However, a notable limitation of this process is its relatively slow inference speed, which inherently restricts its deployment in real-time applications.

Freeze, proposed by Caraffa et al. [60], utilizes frozen alignment as its core. During the training phase, only a lightweight adapter is optimized to align the features of the self-supervised 3D network with the frozen CLIP image features in the same metric space. In inference, RGB-D inputs pass through two frozen backbones separately, and then a lightweight Transformer is used to fuse and vote for 3D keypoints. Since the entire backbone is frozen, training only requires geometric consistency loss. Under zero-shot conditions, the rotation error remains small on occlusion datasets, and it can run at 30 fps on edge GPUs. This method is suitable for fast verification of new objects in scenarios with multiple types of objects, but not in real time.

Lee et al. [61] proposed the UDA-COPE framework, which adapts the network trained in the synthetic domain (CAMERA) to the real domain and introduces multimodal UDA, pseudo-labeling, pose aware bidirectional filtering into category-level pose estimation. The method adopts bidirectional filtering, progression from rough pose estimation and NOCS alignment to bidirectional cleaning, and is universal and easy to replace.

Category-level pose estimation methods are mainly evaluated using the REAL275 dataset. Table 2 summarizes the performance of representative category-level methods on the REAL275 dataset. Specifically, these quantitative evaluations predominantly rely on the 5°2 cm metric to strictly assess rotational and translational accuracy and on the IoU_75 metric to measure the 3D bounding box overlap.

### 3.3. Unseen-Object Methods

Unseen object pose estimation refers to the task of predicting the 6DoF pose of a novel instance or an entirely new object category that was strictly absent during the training phase. The core challenge lies in the fact that unseen objects lack corresponding annotations or CAD models for direct matching. The mainstream approach divides the problem into two steps: first, discovering the object in a new scene and extracting it, and then inferring the 6D pose without real pose labels. Based on their reliance on prior geometric knowledge, deep learning methods for unseen object pose estimation can be broadly classified into two major paradigms: CAD model-based methods and CAD model-independent methods.

#### 3.3.1. Methods Based on CAD Models

**Figure 5:**
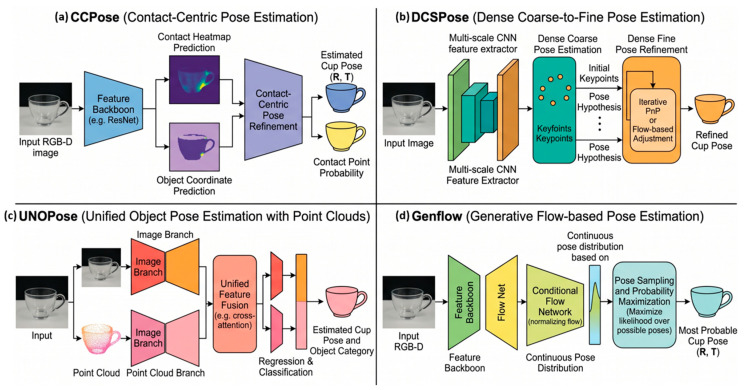
Some of the CAD model-based methods. (**a**) The architecture of CCPose. (**b**) The architecture of DCSPose. (**c**) The architecture of UNOPose. (**d**) The architecture of Genflow.

Although the broader paradigm of unseen object pose estimation typically aims to operate without predefined 3D models, a specialized subset of methods strategically utilizes CAD models as vital prior knowledge during inference. Primarily grounded in feature matching and template matching, the major advantage of these approaches lies in their ability to exploit precise geometric priors, thereby achieving highly accurate and robust pose alignment even for novel instances. However, a significant limitation of this reliance is the strict prerequisite of obtaining a high-quality CAD model at test time, which inherently constrains deployment scalability and flexibility in truly unconstrained, “in-the-wild” environments. As illustrated in Figure 5, several classic methods successfully exemplify the structural pipelines of this paradigm.

The method based on feature matching typically involves designing a network to match features between CAD models and query images, establishing 2D-3D or 3D-3D correspondence, and solving for pose using the PnP algorithm or least squares method. Deng et al. [62] proposed Pos3R, which achieves robust pose estimation of unseen objects without the need for additional training. Using CAD models, render templates from different directions and establish 2D-2D correspondences between test images and templates using the 3D-consistent features generated by MASt3R. Finally, the PnP RANSAC algorithm is used to estimate the 6D pose of the object through 2D-3D correspondences.

Peter et al. [63] proposed CCPose, an innovative framework that successfully estimates the 6D pose of objects from multi-view RGB images by combining machine learning with classical optimization techniques. Firstly, a fully convolutional neural network is used to predict the center and curvature heatmap of an object, and the 3D center point of the object is determined through triangulation of the multi-view center heatmap. Then, using rendering and comparison methods, the 6D pose of the object is optimized by comparing the rendered curvature image with the predicted curvature heatmap. A primary contribution and major advantage of this hybrid pipeline is its exceptional accuracy and physical consistency; by leveraging multi-view constraints, it effectively resolves the depth and scale ambiguities that severely plague single-view estimation.

UNOPose, proposed by Liu et al. [64], standardizes the object representations by constructing SE (3) invariant global reference frameworks (GRFs) and local reference frameworks (LRFs), effectively handling pose and size changes. UNOPose has validated its superior performance on multiple real-world datasets, providing a new solution for pose estimation of unseen objects.

The template matching method utilizes the rendering templates of CAD models for retrieval, and the initial pose is obtained based on the most similar template, which is further refined by a refiner. Yue et al. [65] proposed DCSPose, a dual-channel twin network framework. DCSPose adopts a twin network structure and contrastive learning method to enable the model to quickly adapt to new objects and reduce dependence on annotated data.

Moon et al. [66] proposed the Genflow framework, which strategically guides optical flow learning through pose-induced flow, ensuring that network predictions conform to 3D structures. It then split the confidence level into certainty and pose sensitivity to improve robustness to occluded and texture-sparse areas. A major advantage of this method is its significantly enhanced robustness when handling heavy occlusions and texture-sparse regions. LocPoseNet, proposed by Zhao et al. [67], is used to robustly learn the position prior of unseen objects and, through efficient multi-scale template matching and decoupled parameter prediction, it achieves accurate localization of unseen objects, thereby improving the accuracy of attitude estimation. While the primary contribution of LocPoseNet lies in its exceptional localization precision and efficiency, a notable drawback is its inherent reliance on template matching. This dependency can limit scalability due to the need for extensive template databases.

#### 3.3.2. CAD Model-Independent Methods

In addition to CAD model-based approaches, a rapidly growing body of research focuses on methods that strictly eliminate the prerequisite of possessing an exact object CAD model. Instead of relying on explicit 3D models, these innovative approaches explore alternative priors and representations. Broadly, they can be categorized into several primary streams, including diffusion model-based techniques, geometric constraint-based methods, and voxel-based volumetric representations, among others.

Jiang et al. [68] proposed UnPose, which utilizes a diffusion model to generate multi-view images with pixel-level uncertainty. Through uncertainty-guided fusion and optimization, the pose and reconstruction accuracy are gradually improved. It is mainly divided into four modules: initialization, 3DGS mapping, attitude estimation, and backend optimization. Zhou et al. [69] proposed the PoseDiffusion framework, which uses a dual-branch Transformer feature extraction module and a diffusion model for pose generation and optimization. After three stages of rough pose estimation, pose generation and optimization, and pose refinement (DMPR), the diffusion model refinement significantly reduces the pose errors in this framework. Tang et al. [70] proposed a sparse keypoint representation method called Diff-COPE, which uses learnable keypoints to represent the shape of objects. The diffusion model is used to perform denoising optimization under class conditions, achieving a sparse representation with strong intra-class generalization ability. Combining geometric semantic feature fusion, this framework effectively solves the problems of large intra-class differences and poor generalization in category-level pose estimation. Liu et al. [71] proposed the HIPPo framework, which uses the image-to-3D prior of a diffusion model to generate a complete 3D mesh from a single view of an object and continuously optimizes it online, while achieving high-precision 6D pose estimation. A significant advantage of this method is that it consistently outperforms traditional Structure-from-Motion (SfM) and CAD-based approaches, particularly demonstrating exceptional robustness in highly challenging scenarios.

Keunhong et al. [72] proposed the LatentFusion framework, which constructs a latent 3D voxel representation and optimizes the structure during the inference phase through a differentiable renderer for rendering-based comparison to directly estimate the pose. It achieves SOTA performance on multiple datasets without the need for 3D models or retraining. Chen et al. [73] proposed a geometric constraint learning method and proposed the Geo6D mechanism based on the RPF formula, which is used to construct a relative offset representation in a 2D normalized image plane. The pose transformation and perspective projection constraints are retained, and the absolute pose distribution is decoupled to solve the problem of the pose distribution gap. This method is suitable for small sample (3–16 frames) scenes and is end-to-end differentiable, but there is ambiguity in the rotation of symmetric objects, and the speed is relatively slow.

Cai et al. [74] pioneered the integration of 3D Gaussian Splatting into 6D pose estimation with their proposed GS-Pose framework. The semantic detector directly segmented the object, followed by template matching to provide the initial 6D pose. Then, GS Refiner was used to iteratively optimize the rendering comparison process, resulting in sub-pixel-level refined poses. The primary contribution and major advantage of this framework is its ability to achieve sub-pixel-level pose refinement, yielding exceptional accuracy. Zhao et al. [75] proposed DVMNet++, which is the first voxel-level, hypothesis-free, end-to-end relative pose network. It uses an HS-language visual detector and DINOv2 retrieval to automatically obtain reliable frames, approximates the 2D center with the frame center, and combines the camera intrinsic parameters to obtain the relative pose. A significant advantage of DVMNet++ is its strong applicability to industrial parts using solely RGB references, effectively eliminating the need for complex 3D CAD models. This method is suitable for industrial parts with only RGB references, but it has a relatively general effect on outdoor large scenes and small objects at long distances.

Cao et al. [76] also introduced 3D Gaussian Splatting iterative rendering into model-free 6DoF pose estimation, thereby proposing iG-6Dof. This framework treats 3DGS as a differentiable renderer, using group features for rough estimation and iterative refinement, with high accuracy and fast speed, but sensitive to reference viewpoint density. This framework achieves pixel-level refinement without models and densely samples on SO (3) to solve the problem of sparse reference viewpoints. This method is suitable for small- or zero-sample indoor scenes. It is optimal for weakly textured objects, but the lack of depth information causes scale ambiguity and ambiguity in symmetrical objects.

In summary, the diverse pose estimation methods reviewed in this paper exhibit distinct structural advantages and inherent limitations, indicating that their effectiveness varies significantly depending on the specific object characteristics and environmental scenarios. Table 3 summarizes the quantitative performance metrics of several representative methods discussed in this article on the LM-O and YCB-V datasets. It should be noted that some data entries are omitted, as certain methods have not been officially evaluated on these specific benchmarks.

## 4. Datasets

The performance of 6-degree-of-freedom pose estimation highly depends on the quality and diversity of the dataset. The annotation accuracy, scene coverage, object categories, and other factors of the dataset directly affect the generalization ability and robustness of the model. This chapter will systematically review the methods for organizing the 6DoF attitude estimation dataset, including selection criteria, preprocessing, and annotation specifications. The publicly available datasets commonly used for 6D pose estimation can be classified into two main categories: datasets for instance-level methods and datasets for category-level methods.

### 4.1. Datasets for Instance-Level Methods

LineMod (LM) [77] is a classic dataset for pose estimation, containing 15 everyday objects such as cam, can, cat, driller, etc. Each object has approximately 1.2 k RGB-D images, totaling about 18 k frames. These objects have significant differences in shape but generally lack texture. The scene is simple, usually consisting of a desktop and a black screen, with limited lighting variation and light occlusion/stacking. This dataset is suitable for verifying the basic ability of methods based on corresponding points, templates, and deep learning under the condition of weakly textured objects, and is also one of the core subsets of BOP challenges.

LineMod-Occluded (LMO) [78] selected only eight objects, such as can, cat, duck, etc., from LM, and additionally annotated 1214 severely occluded frames in the original sequence as the test set. This dataset has the same CAD model as LM, so the model can be migrated at zero cost, and the robustness of occlusion can be measured by simply changing the testing protocol. All frames are in high-intensity occlusion environments such as multi-object stacking and hand–object interaction. This dataset specifically examines the recall rate of algorithms under the triple difficulties of occlusion, symmetry, and weak texture and is a mandatory test item in the difficulty-level of BOP.

T-LESS [79] contains 30 types of industrial parts, such as switches, flanges, and brackets, with 1296 RGB-D images per type, totaling approximately 39 k frames, and an additional 3.6 M PBR composite images are provided for training enhancement. These objects have almost no texture, similar colors, highly similar shapes, and 11 have continuous rotational symmetry. This dataset is suitable for research on industrial picking, symmetric blurring, and weak texture modeling.

YCB-Video [80] contains 21 household items (taken from the YCB Object Set of Class 50), and a total of 92 RGB-D videos, with a total length of 16 h and 134 k frames. All videos are continuous and can run single-frame estimation or tracking, providing official RGB instance segmentation results and supporting detection pose separation evaluation. This dataset is suitable for home service robots, visual grasping, long-term tracking and multi-object joint estimation.

ITODD [81] contains 28 types of industrial small parts such as screws, gears and interfaces, with less than 1 k publicly available verification drawings. The actual acquisition uses a dual-mode camera with both grayscale and depth. Grayscale images have no color difference information and low depth noise, making them suitable for testing pure geometry or grayscale depth fusion methods. This dataset is suitable for verifying pose estimation algorithms for small-sized industrial parts, high-precision assembly, and objects with no color clues.

### 4.2. Datasets for Category-Level Methods

CAMERA25 [51] and REAL275 [82] are often referred to as the “NOCS series”, which includes six categories of daily necessities such as bottles, bowls, and cameras. CAMERA25 extracted 1085 CAD models from ShapeNet for rendering, with a total of 300 k synthesized RGB-D images and 25 k images left for testing. This dataset is a pure synthetic subset with significant domain differences, which can be used to study the transition from synthesis to reality. REAL275 collected data from 7 real indoor scenes, 4.3 k trained images, and 2.75 k tested images. Three never seen instances are placed for each category, and specific CAD is not provided during testing, relying only on category semantics. There are significant differences between the synthesis and real domain, and REAL275 has a cluttered and occluded background, which is commonly used for domain adaptation.

Wild6D [83] contains five categories: mug, bowl, bottle, remote, and laptop, with a total of 5166 videos, with a total frame rate of 1.1 M. The official team has also released 29 k keyframes with fine pose truth values. This dataset consists of 100% realistic scenes, diverse backgrounds on desktop, floor, sofa, and windowsill. The blockage, lighting changes, and motion blur are stronger than REAL275. This dataset is suitable for real-world scenarios, weakly supervised or self-supervised, long-term tracking, and occlusion robustness verification.

Omni6D [84] is the largest category-level benchmark for big vocabulary to date, containing 4688 real scan instances of 166 categories, 800,000 RGB-D frames, with an average of 4.8 k images per category. This dataset covers 166 categories, including home, tools, office, toys, sports, hardware, etc., with significant differences in shape and material, and a high proportion of symmetrical, reflective, transparent objects. It is suitable for large vocabulary category-level, open-world pose, symmetrical, reflective, transparent materials, and next-generation universal robot grasping. Table 4 shows the overview of the commonly used datasets.

### 4.3. Metrics

The evaluation index for pose estimation uses several rulers with different scales to measure how much the placement position and orientation of the object given by the algorithm differ from the true value. It translates subjective feelings of good or bad into measurable values, allowing us to compare different methods, track progress, or meet the specific business requirements under the same standard.

The most intuitive way to check whether an object is accurately estimated is to place its 3D model in space according to the estimated pose, and then place it again according to the true pose to measure the positional difference between the vertices of the same batch is. That is how ADD [77] works. If an object lacks symmetry, ADD is the gold standard. The threshold is generally set at 10% of the maximum diameter of the object or 2 cm, and if it is less than this value, the pose is considered correct. ADD can be expressed as follows:

(1) $$e_{\mathrm{ADD}}=\frac{1}{\left|V\right|}\sum_{v\in V}||\hat{Rv}+\hat{t}-\left(Rv+t\right)|{|}_{2}$$

where *V* is the 3D model point set, *R* is the rotation matrix, and *t* is the translation matrix.

ADD-S [85] is a symmetric version of average point drift, whose core idea is that symmetric objects allow for multiple correct answers, so instead of using a one-to-one distance, the one-to-many approach takes the minimum. Since 2019, BOP Challenge has designated ADD-S as the default 3D metric because it can accommodate both “asymmetric” and “symmetric” objects simultaneously. It can be expressed as follows:

(2) $$e_{\mathrm{ADD}-S}=\frac{1}{\left|V\right|}\sum_{v\in V}\underset{s\in S}{\min}||\hat{Rv}+\hat{t}-\left(R_{sv}+t_{s}\right)|{|}_{2}$$

where the symbol *s* represents the symmetric set of the objects.

MSSD [86] is the least likely drift. For each vertex *v*, calculate the minimum distance from the estimated position to all symmetric true positions, and then take out the maximum value from these minimum distances. The threshold is usually still 2 cm or 5 mm; as long as the farthest vertex does not exceed the standard, it is judged correctly. MSSD can be represented as follows:

(3) $$e_{\mathrm{MSSD}}=\underset{v\in V}{\max}\;\underset{s\in S}{\min}||\hat{Rv}+\hat{t}-\left(R_{sv}+t_{s}\right)|{|}_{2}$$

where the symbol *s* represents the symmetric set of the objects.

MSPD [86] is the pixel reprojection error. First, all vertices of the object are projected onto the camera imaging plane. For the same vertex, the “pixel coordinates projected from the estimated pose” are compared with the pixel coordinates projected from all symmetric true poses to obtain the shortest distance. Finally, the maximum value among the shortest image distances of all vertices is taken as MSPD. It can be represented as follows:

(4) $$e_{\mathrm{MSPD}}=\underset{v\in V}{\max}\;\underset{s\in S}{\min}||\pi \left(\hat{Rv}+\hat{t}\right)-\pi \left(R_{sv}+t_{s}\right)|{|}_{2}$$

where the symbols *s* and *v*, respectively, represent the symmetric set of the objects and the pixel coordinates projected from the estimated pose.

Intersection over Union (IoU) [87] serves as a standard metric for spatial accuracy in object detection and segmentation tasks. IoU represents the overlap rate between the predicted hypothesis and the corresponding ground truth by computing the ratio of their intersection area to their combined union area. The metric yields a strictly bounded value, where higher scores reflect superior localization performance, culminating in a value of 1.0 for perfect spatial alignment. The IoU mathematical formula is expressed as:

(5) $$\begin{array}{c} IoU=\frac{\left|B_{p}\cap B_{gt}\right|}{\left|B_{p}\cup B_{gt}\right|}=\frac{\left|B_{p}\cap B_{gt}\right|}{\left|B_{p}\right|+\left|B_{gt}\right|-\left|B_{p}\cap B_{gt}\right|} \end{array}$$

where *A* and *B*, respectively, represent the predicted bound box and the ground truth bounding box.

## 5. Applications

Pose estimation technology has deeply penetrated into fields such as intelligent manufacturing, autonomous robot operations, augmented reality, healthcare, and aerospace, and has become a key support for achieving high-precision perception, automated operations, and virtual reality integration. The following is a systematic academic exposition on core application scenarios, technology adaptation logic, industry value, and cutting-edge trends.

### 5.1. Robot Automation

Bin-picking remains one of the most demanding and challenging tasks for industrial robotic manipulation [88]. Target objects often exist in the bin in a stacked, occluded, arbitrary pose and weakly textured state. A fundamental limitation of traditional template matching and 2D vision-based methods is their complete inability to handle such complexity, as they inherently lack crucial depth and spatial orientation information. The primary contribution and major advantage of this modern approach is its capability to achieve robust, real-time pose calculations for single or multiple objects, even in highly cluttered scenes. By seamlessly integrating RGB-D data, multi-view geometry, geometric contours, and 3D model priors, these advanced methods provide highly accurate grasping poses for robotic end-effectors, effectively solving the core engineering challenge of reliably extracting targets from disorganized bins.

In the field of precision assembly [89] and robot collaborative assembly, precision assembly of automobiles, aviation, and 3C electronics requires millimeter-level or even sub-millimeter-level pose accuracy. The assembly objects are mostly non-textured metal or composite parts (such as engine cylinder blocks, gearbox housings, aircraft skins, and mobile phone frames), and the assembly process requires real-time perception of the relative pose of the parts and dynamic adjustment.

In terms of automated quality inspection and 3D measurement, industrial quality inspection requires non-contact measurement of the dimensional accuracy, form and position tolerances, assembly clearances, and surface defects of weakly textured parts [90]. 6DoF pose estimation is a prerequisite for achieving precise alignment between the measurement coordinate system and the part coordinate system, including the positioning and gap measurement of welding points on the white body of automobiles, the detection of flying edges and cracks in metal castings, the thickness and flatness detection of transparent glass and plastic parts, and the three-dimensional morphology measurement of aircraft engines. Both pose estimation and structured light 3D reconstruction, laser line scanning, and binocular vision fusion are required. Firstly, the 6DoF pose of the part is calculated, and then the measurement data is converted to the local coordinate system of the part to achieve high-precision size comparison and defect detection.

In the field of remote sensing, SpectralGPT [91] fills the gap in the field of spectral remote sensing that lacks dedicated basic models, achieving efficient utilization of massive heterogeneous spectral data. It can use specific band combinations to evaluate atmospheric conditions and assist in atmospheric correction of remote sensing images.

### 5.2. Healthcare

In medical settings, a large amount of non-textured or low-textured human tissues, implants, and surgical instruments require 6DoF pose estimation to provide spatial positioning support for minimally invasive surgery and image-guided therapy. Minimally invasive surgical robot navigation, laparoscopic, orthopedic, and neurosurgical minimally invasive surgeries rely on real-time pose perception of surgical instruments and human tissues by robots. Surgical instruments are mostly weakly textured or reflective objects, while human tissues have sparse textures [92].

There is also medical imaging guidance and prosthesis positioning. In joint replacement, spinal correction, and other surgeries, 6DoF pose estimation is required for metal prostheses and bones to achieve accurate matching between prostheses and bones. Real-time pose estimation during surgery is used to obtain the relative posture between the bone and the prosthesis, guiding doctors to adjust the position of the prosthesis and improve the success rate of surgery and patient prognosis.

Li et al. [93] proposed Im2State, a novel hyperspectral–multispectral image fusion framework based on a state space model. The im2state framework reconstructs high-resolution hyperspectral images by fusing low-resolution HSI and high-resolution MSI. In medical scenes, this means that high-spatial-resolution tissue images can be obtained while preserving the fine spectral information required for diagnosis. At the same time, it is helpful to identify the micro-lesion structure, and improve the accuracy of diagnosis and surgery.

### 5.3. Aerospace

Structural components in the aerospace field, such as aircraft skins, engine blades, and satellite parts, are mostly made of weakly textured metals or composite materials, and require extremely high processing or assembly accuracy. Unmanned platforms in the field of national defense need to perform pose perception on weakly textured targets such as armored vehicles, missiles, and drones.

The processing and assembly of large weakly textured structural components such as aircraft skins, wings, and fuselage frames require real-time perception of the part pose to compensate for processing errors and deformations [94]. A fusion scheme of laser tracker and visual pose estimation is adopted to globally locate and locally optimize the pose of large weakly textured parts, meeting the requirements of micrometer-level assembly accuracy. Drones, unmanned vehicles, and unmanned ships need to perform 6DoF pose estimation on weakly textured targets in complex scenes, such as armored vehicles, ships, and low-altitude aircraft, to achieve target tracking, strike guidance, and obstacle avoidance.

### 5.4. Augmented Reality and Virtual Reality

The core of AR/VR is the precise alignment of virtual content with the real world. Weakly textured objects, such as walls, desktops, metal devices, and transparent objects are the main components of the real environment, and their 6DoF pose estimation is the key to achieving stable and high-precision virtual–real fusion [95].

Industrial AR reduces operational difficulty and error rates by overlaying virtual guidance information on real weakly textured devices such as machine tools, engines, and production lines [96]. The strong reflection, low lighting, and dynamic occlusion in industrial environments lead to AR tracking drift, requiring the use of edge feature SLAM, multimodal sensor fusion, and attitude optimization to enhance stability.

Consumer-grade AR [97], such as mobile AR and AR glasses, requires 6DoF pose estimation for weakly textured indoor surfaces, furniture, and appliances to achieve virtual object placement, interaction, and spatial navigation. Adopting lightweight deep learning models, such as edge pose regression, plane detection, and pose optimization, will help achieve real-time, low-power pose tracking of weakly textured objects on mobile devices.

## 6. Conclusions

This review has systematically examined both classical and recent methods for pose estimation of weakly textured objects, offering a comprehensive comparative analysis of their respective strengths and limitations, as well as an overview of their practical applications. Despite notable advances, several fundamental challenges persist: the scarcity of annotated real-world data, the difficulty of achieving high-precision estimation under adverse conditions, and the prohibitive computational cost of deploying large-scale models on resource-constrained platforms. Building upon these challenges, we identify several promising research directions that we anticipate will shape the trajectory of the field.

Bridging the Synthetic-to-Real Domain Gap. The prohibitive cost of manually annotating large-scale real images with accurate 6D pose labels has made synthetic data indispensable. However, the existing domain gap between rendered and real imagery remains a central bottleneck. A particularly promising avenue to address this lies at the intersection of generative foundation models and 3D Gaussian Splatting (3DGS). In this approach, a single real RGB-D observation can serve as a seed from which multi-view diffusion models synthesize a continuous stream of novel viewpoints, while 3DGS-based implicit reconstruction subsequently filters out geometrically inconsistent or uncertain regions. This self-generating pipeline yields large-scale, densely annotated synthetic streams with soft supervision signals, offering a scalable and sustainable solution to real data scarcity that circumvents the need for labor-intensive manual annotation.

Robust Estimation Under Geometric Ambiguity and Occlusion. Weakly textured surfaces present inherently ambiguous RGB signals; however, their surface normals and depth profiles frequently remain continuous and structurally informative. For transparent and low-reflectance objects—where both photometric and depth cues are unreliable—future research should explore the complementary use of multi-view geometry, polarization imaging, depth completion networks, and surface normal estimation to enrich the geometric representations. Occlusion handling represents perhaps the most pressing open problem in the field. Existing methods degrade sharply under severe or unpredictable occlusion patterns, and no general-purpose solution has yet emerged. Promising directions include diffusion model-based multi-hypothesis pose generation, graph neural networks for local feature propagation across partially observed surfaces, and part-to-part correspondence strategies that remain robust to incomplete inputs. A particularly compelling emerging paradigm explicitly embeds depth-guided normal regularization into the training objective. In this approach, lightweight sequence models, such as Mamba or state space models (SSMs), first perform depth completion and denoising. Subsequently, normal–depth consistency constraints are incorporated into the loss function, enabling geometric supervision to flow directly into the pose regression branch. Complementarily, locally sensitive hash attention mechanisms can dynamically up-weight geometric features in occluded regions, mitigating the pose ambiguity that arises from the absence of texture.

Toward Efficient, End-to-End Deployable Systems. Contemporary high-accuracy models typically entail large parameter counts and substantial computational overhead, rendering their direct deployment on mobile devices and robotic endpoints impractical. A central design imperative for future work is the development of unified, end-to-end architectures that jointly perform object detection, instance segmentation, and pose estimation within a single differentiable network. This eliminates the reliance on non-differentiable solvers, such as PnP, as post-processing steps, and substantially reduces both inference latency and system complexity. As depth sensors remain absent from the majority of consumer and mobile platforms, monocular RGB-based methods, potentially augmented by large vision foundation models for self-supervised depth estimation, will become increasingly important. Established model compression techniques, including knowledge distillation, structured pruning, and neural architecture search, provide complementary pathways to reduce parameter counts and inference time while preserving accuracy to a practical degree.

Open Problems and Outlook. Beyond the directions outlined above, several fundamental bottlenecks remain unresolved: (i) achieving category-level generalization to novel instances without dependency on exact CAD models; (ii) ensuring robust pose estimation under extreme viewpoint variation and severe domain shift; and (iii) systematically embedding uncertainty quantification into pose predictions, which is critical for safe robot manipulation. Ultimately, resolving these challenges necessitates stepping beyond isolated algorithmic improvements. We anticipate that a tight integration of large vision–language models, differentiable rendering, and efficient geometric deep learning will significantly drive the forthcoming paradigm shift, substantially elevating the state of the art in weakly textured object pose estimation in the coming years.

## Figures and Tables

**Table 1:** Representative instance-level methods. We report the average recall of ADD(s) within 10% of the object diameter of LM and LM-O datasets, and the AUC of ADD-S (<0.1 m) on the YCB-V dataset.

Type	Model	Published Year	Input	Inference Time	LM-O ADD(s)	LM ADD(s)	YCB-V ADDs
Instance-level methods	PoseCNN	2018	RGB, CAD	90 ms	76.1	76.9	78.9
	DeepIM	2018	RGB, CAD	100 ms	88.7	89.1	92.4
	DPOD	2019	RGB, CAD	12 ms	32.8	82.9	87.5
	INeRF	2020	RGB	-	-	-	-
	PVNet	2019	RGB, CAD	40 ms	89.6	97.3	95.8
	ZebraPose	2022	RGB, CAD	35 ms	71.2	96.3	86.9
	DenseFusion	2019	RGB-D, CAD	60 ms	-	94.9	92.1
	PVN3D	2020	RGB-D, CAD	200 ms	87.4	97.6	94.2
	Transpose	2023	RGB-D, CAD	50~80 ms	86.5	96.8	92.8
	SAM-6D	2024	RGB-D, CAD	1 s	91.3	-	-
	PCRNet	2019	Point cloud, CAD	-	84.3	95.5	89.6
	PointPoseNet	2020	Point cloud, CAD	-	82.6	94.8	88.1
	DeepGMR	2020	Point cloud, CAD	-	85.7	96.1	91.9
	PS6D	2024	Point cloud, CAD	-	-	98.1	-

**Table 2:** Representative category-level methods. For each method, we report its 5 properties, the published year, input modality, inference time, and the performance metrics. We report the 5°2 cm metric and the IoU_75 metric of the REAL275 dataset.

Type	Model	Published Year	Input	Inference Time	5°2 cm	IoU_75
Category-level methods	NOCS	2019	RGB-D	100 ms	7.2	30.1
	HS-Pose	2025	Point cloud, CAD	45 ms	61.7	71.5
	GenPose	2023	RGB	>800 ms	89.5	88.1
	SpotPose	2025	RGB-D	50 ms	62.0	71.2
	ZeroPose	2023	RGB, CAD	150 ms	48.1	-
	SecondPose	2024	RGB, point cloud	65 ms	56.2	49.7
	OnePose++	2023	RGB	-	65.3	33.1
	AG-Pose	2024	RGB-D	35 ms	57.0	69.5
	IST-Net	2023	Point cloud	25 ms	54.6	70.3
	UDA-COPE	2021	Point cloud	60 ms	30.4	62.5
	TSM-Pose	2026	RGB-D	40 ms	62.4	72.8

**Table 3:** Representative unseen-object methods. We report the BOP-M across the LM-O and YCB-V datasets for various methods. For the convenience of readers’ comparison, we also report the input modes and the inference time of different methods. However, the inference time is only for reference, as the performance of different devices differs.

Type	Model	Published Year	Input	Inference Time	LM-O ADD(s)	YCB-V ADDs
Unseen-object methods	CCPose	2021	RGB-D, CAD	800 ms	-	-
	UNOPose	2024	RGB-D, CAD	-	70.5	82.9
	DCSPose	2024	RGB-D, CAD	-	65.3	82.1
	Genflow	2024	RGB-D, CAD	50 ms	62.9	82.5
	UnPose	2025	RGB-D	2 s	86.8	85.3
	Diff-COPE	2024	RGB-D	-	78.2	81.5
	HIPPo	2025	RGB-D	75 ms	92.9	97.0
	LatentFusion	2020	RGB-D	500 ms	38.5	74.3
	Geo6D	2022	RGB-D	30 ms	79.8	91.6
	GS-Pose	2024	RGB	200 ms	-	40.1
	DVMNet++	2025	RGB	80 ms	-	-
	iG-6Dof	2024	RGB-D	200 ms	74.8	41.2

**Table 4:** Overview of the datasets for the evaluation of object pose estimation.

Dataset	CAD Model	Image Modality	Category	Frame	Feature	Application
LineMod	CAD	RGBD	15	18 k	Daily objects	Corresponding points and templates
LineMod-Occluded	CAD	RGBD	8	9.6 k	Occlusion	Occlusion scene
T-LESS	CAD	RGBD	30	39 k	Almost no texture	Industrial picking
YCB-Video	CAD	RGBD	21	134 k	Continuous videos	Home service robots
ITODD	CAD	Grayscale-D	28	1 k	Industrial small parts	High-precision pose estimation
CAMERA25	CAD	RGBD	6	325 k	Pure synthetic	Synthesis to reality
REAL25	No CAD	RGBD	6	7 k	Realistic scene	Domain adaptation
Wild6D	No CAD	RGBD	5	1.1 M	Blockage	Weakly supervised
Omni6D	No CAD	RGBD	166	800 k	Big vocabulary	Large vocabulary

## Data Availability

No new data were created or analyzed in this study. Data sharing is not applicable to this article.
